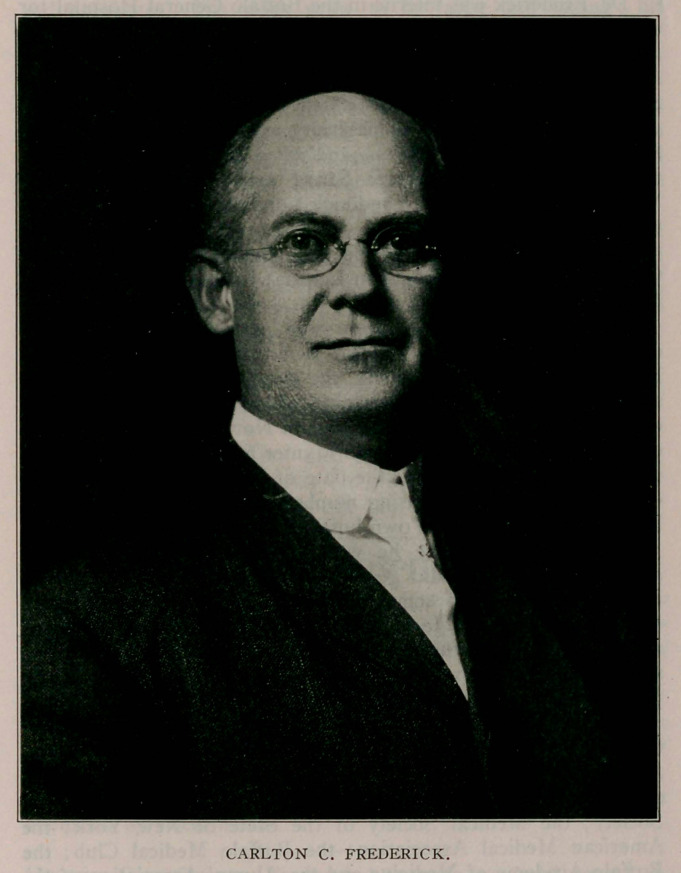# Carlton C. Frederick

**Published:** 1911-06

**Authors:** 


### OBITUARY

---

Carlton C. Frederick, one of the best known surgeons of Western New York, died on April 30 at his home, 64 Richmond Avenue, Buffalo, after a brief illness. Funeral services were held from the First Presbyterian Church, on May 3.

Dr. Frederick is survived by his wife and two children, Thomas Lothrop Frederick and Ruth Frederick.

At the time of his death Dr. Frederick was president of the Buffalo Academy of Medicine and a special memorial meeting was held on May 2, at which resolutions were passed and addresses made by Dr. James W. Putnam, Dr. Matthew D. Mann, Dr. John Pryor, Dr. Stephen Y. Howell and Dr. Earl Lothrop.

The committee on draughting a memorial to Dr. Carlton C. Frederick, president of the Buffalo Academy of Medicine reported as follows:

The officers and fellows of the Buffalo Academy of Medicine, having learned with deep regret of the death of their honored president, Dr. Carlton C. Frederick, desire to give expression to their sorrow in the loss of an honored colleague and to their high appreciation of Dr. Frederick as a man and as a physician and surgeon.

CARLTON C. FREDERICK.

Dr. Frederick was born in Hamburg, N. Y., May 1, 1855, and spent his early life at his birth-place. When he was fourteen years of age his parents moved to Buffalo and he lived with them on the southeast corner of Allen and Park streets. He was educated at the public schools and the Central High School and graduated from the University of Michigan, Ann Arbor, with the degree of B.S. in 1877. He taught school at North Evans, N. Y., in 1877 and 1878, and in Professor Horace Brigg's Classical School in 1878 and 1879. In 1878 he entered the University of Buffalo and graduated in medicine in 1881.

Dr. Frederick was interne in the Buffalo General Hospital for one year after his graduation, and assisted Dr. M. D. Mann who, at that time introduced in Buffalo modern methods in abdominal surgery. He was obstetrician to St. Mary's Asylum on Edward Street in 1885. He was associated with Dr. Thomas Lothrop when the latter started a maternity hospital at the corner of Seventh and Maryland streets, and when Dr. Lothrop purchased the property at 191 Georgia Street and founded the Buffalo Woman's Hospital. Dr. Frederick purchased a half interest in the hospital at a later date.

In 1891 he went to Europe and studied under Martin and Olshausen in Berlin; Sanger in Leipsic; Leopold in Dresden and Lawson Tait in England.

The first abdominal operation was performed by Dr. Frederick in the Woman's Hospital November 19, 1891, the case being one of double pyosalpinx, the late Dr. W. S. Tremaine assisting. The second intraabdominal operation done by Dr. Frederick in the Woman's Hospital was on November 21, 1891,—two days later, the late Drs. Herman Mynter and Jacob Meyer assisting. From that time until the date of his last illness Dr. Frederick performed an increasing number of difficult, successful and brilliant operations in his own hospital and elsewhere.

In his early practice he was a remarkably successful obstetrician and a splendid general practitioner. He had one of the largest obstetric practices in Buffalo and was unusually successful. His knowledge of disease grew large and valuable and when, in later years, he devoted himself to abdominal and pelvic surgery his early experience gave him a finely tempered and rare judgment.

Dr. Frederick was president of the Medical Association of Central New York in 1908. He was a member of the Alumni Association of the University of Michigan; of the alumni association of the University of Buffalo; of the Erie County Medical Society; the Medical Society of the State of New York; the American Medical Association; the Buffalo Medical Club; the Buffalo Academy of Medicine and the Alumni Association of the Buffalo General Hospital. He was also a member of the American Gynecological Society and the American Association of Obstetricians and Gynecologists. He was adjunct professor of obstetrics in the medical department of the Niagara University and later clinical professor of gynecology in the University of Buffalo. He made many valuable contributions to medical journals.

Dr. Frederick had a cheerful, buoyant nature and was energetic and optimistic in his work. He was a skilful diagnostician and a resourceful, rapid and able operator. His results were excellent. He was kind and helpful and was beloved by his patients and associates. His work grew to be large and taxing; his reputation was widespread and he was sought much in consultation. He was frequently called upon to operate in the country and in neighboring and distant towns and cities. With splendid willingness and kindness he responded to many overtaxing demands upon his time, strength and skill. He was deeply interested in and helpful to the young physicians. He showed a big-hearted concern and humanitarian interest that endeared him to a large clientele and wide circle of friends and acquaintances.

In the death of Dr. Frederick, the city of Buffalo has lost a valuable citizen and the profession of medicine a distinguished and brilliant member. It therefore seems highly appropriate that we should adopt this memorial to serve as a record of our pride in our late colleague and of our sorrow at his loss.

## Figures and Tables

**Figure f1:**